# Kunxian capsule alleviates podocyte injury and proteinuria by inactivating β-catenin in db/db mice

**DOI:** 10.3389/fmed.2023.1213191

**Published:** 2023-06-30

**Authors:** Bo Jin, Jing Liu, Yan Zhu, Jian Lu, Qingyan Zhang, Yan Liang, Qiuyuan Shao, Chunming Jiang

**Affiliations:** Department of Nephrology, Nanjing Drum Tower Hospital, The Affiliated Hospital of Nanjing University School of Medicine, Nanjing, Jiangsu, China

**Keywords:** Kunxian capsule, diabetic kidney disease, podocyte, β-Catenin, epithelial-to-mesenchymal transition

## Abstract

**Background:**

Diabetic kidney disease (DKD) remains the primary cause of end-stage renal disease (ESRD) globally, but treatment options are limited. Kunxian capsule (KXC) has been utilized for the treatment of autoimmune diseases and IgA nephropathy in China. However, its effect on DKD remains poorly investigated. Therefore, this study aimed to explore the protective effect of KXC in db/db mice and elucidate its underlying mechanism.

**Methods:**

The renoprotective effects of KXC were assessed in a DKD mouse model using male BKS db/db diabetic mice. After 8 weeks of treatment, the urinary albumin-to-creatinine ratio (UACR), blood biochemical parameters, renal histopathological manifestation, and podocyte ultrastructural changes were evaluated. Additionally, the expression of podocyte epithelial-to-mesenchymal transition (EMT) markers [WT1, ZO-1, and collogen I (Col1a1)] was quantitatively analyzed. Furthermore, we explored the role of KXC in the β-catenin signaling pathway to elucidate the underlying mechanism of KXC’s renoprotective effect.

**Results:**

KXC treatment effectively reduced albuminuria and attenuated renal structural abnormalities in db/db mice. Additionally, KXC restored the protein and mRNA expression of WT1 and ZO-1 while suppressing the expression of Col1a1 in db/db mice, indicating its ability to alleviate podocyte EMT. Mechanistically, KXC exerted a significant suppressive effect on the activation of β-catenin signaling in diabetic kidneys.

**Conclusion:**

KXC has the potential to protect podocytes during DKD by alleviating podocyte EMT through inactivating β-catenin signaling.

## Introduction

DKD is a common and severe complication in diabetic patients and is characterized by excessive urinary albumin excretion and progressive renal function impairment. DKD remains the primary cause of ESRD worldwide, but therapeutic options are limited (1–3). Over the past three decades, renin-angiotensin system (RAS) inhibitors have consistently served as the mainstay treatment for slowing the progression of DKD. However, the renoprotection provided by these drugs may be insufficient, particularly in advanced stages of DKD (4, 5). In recent years, as our understanding of the underlying pathogenic mechanisms of DKD has advanced, novel drugs such as sodium-glucose transport protein-2 inhibitors (SGLT-2i), endothelin antagonists, mineralocorticoid receptor antagonists (MRAs) and glucagon-like peptide (GLP)-1 agonists have emerged as potential treatments for improving DKD outcomes. Despite their promising benefits in clinical practice, the risk of progressive kidney function decline and the development of ESRD remains substantial (6, 7). Therefore, more effective therapeutic strategies are urgently needed to prevent and treat DKD.

Traditional Chinese medicine (TCM) has a longstanding history of use for treating diabetes and DKD (8–11). Kunxian capsule (KXC), a patent TCM prescription, is composed of four medicinal herbs: *Tripterygium hypoglaucum Hutch*, *Cuscuta chinensis Lam*, *Epimedium brevicornu Maxim*, and *Lycium barbarum L*. In China, KXC has gained widespread popularity for the treatment of autoimmune diseases due to its notable immunomodulatory and anti-inflammatory activities (12–14). Previous clinical studies have demonstrated that KXC has low toxicity and is a safe treatment option (15). Furthermore, accumulating clinical data from small cohorts have indicated that KXC can attenuate proteinuria and impede the progression of IgA nephropathy (16). Notably, a randomized clinical trial is currently ongoing to verify the renoprotective effect of KXC in individuals with DKD (17). However, the protective role and potential mechanism of KXC in DKD remain obscure. Therefore, this study aimed to explore the protective effects of KXC on DKD and elucidate its underlying mechanism.

## Materials and methods

### Animal experiment

Male BKS db/db diabetic mice and age-matched db/m mice (Nanjing University Model Animal Research Center) were used in the study. The mice were 8 weeks old and housed in a specific pathogen-free (SPF) environment at our animal center. Diabetic db/db mice with random blood glucose levels exceeding 16.7 mmol/L were included in the observational studies. Subsequently, the mice were randomly assigned to three groups (*n* = 6 each): db/m group, db/db group, and db/db + KXC group. KXC, provided by Guangzhou Chen Li Ji Pharmaceutical Co. Ltd (Guangzhou, China), was dissolved in 0.5% carboxymethylcellulose sodium (CMC-Na) to increase its solubility before use. According to clinical practice and previous literature (18), the db/db + KXC group received intragastric administration of KXC (234 mg/kg/day). The db/m and db/db groups were administered the same volume of vehicle (0.5% CMC-Na) by gavage. Samples of spot urine were collected every 4 weeks during the treatment and stored at −80°C. After 8 weeks of treatment, fasting blood glucose (FBG) was measured in each group using a glucometer (LifeScan Milpitas, CA, United States) with blood samples obtained from the mouse tail vein. Subsequently, the mice were sacrificed, and blood samples, as well as kidney tissues, were harvested.

The Ethics Committee on Animal Experiments of Nanjing Drum Tower Hospital (Nanjing, China) approved all experimental protocols in accordance with the guidelines established by the Committee on Laboratory Animals.

### Urine and blood examination

Albumin and creatinine levels in urine were measured using the Albumin ELISA Kit (GeneTex) and QuantiChrom™ Creatinine Assay Kit (BioAssay Systems), respectively. Blood specimens were collected *via* cardiac puncture. The levels of serum creatinine (Scr), alanine transaminase (ALT), and aspartate transaminase (AST) were analyzed using an automatic biochemical analyzer (Hitachi).

### Histological and Immunohistochemical analysis

The renal tissues were excised, fixed in 4% PFA and embedded in paraffin wax. Subsequently, renal tissues were sectioned into 4-μm slices. To assess glomerular matrix accumulation, slides were stained with periodic acid-Schiff (PAS). For immunohistochemical staining, slides were deparaffinized, and then blocked with 10% BAS at room temperature. Tissues were incubated overnight at 4°C with primary antibodies against WT1 (Abcam Cat# ab89901, 1:100), ZO-1 (GeneTex Cat# GTX636399, 1:100), Col1a1 (Santa Cruz Ca# sc-25,974, 1:100), β-catenin (Abcam Ca# ab32572, 1:100), and snail1 (Abcam Ca# ab53519; 1:100). The sections were then incubated with secondary antibodies for 1 h. Color development was achieved using diaminobenzidine (DAB). Histological changes in the tissue were examined using a light microscope (Nikon E800 microscope).

### Transmission electron microscopy

Transmission electron microscopy (TEM) was performed to examine the ultrastructural changes in podocytes. The renal cortex was sectioned into 1 mm^3^ pieces, which were then fixed in 3.75% glutaraldehyde for 4 h. Subsequently, the specimens were treated with 1% osmic acid for 2 h. After gradual dehydration of tissues in acetone and ethanol, the specimens were embedded in epoxy resin (SPI, Indianapolis, IN, United States). Ultrathin slices were stained with uranyl acetate and lead citrate. GBM and podocytes were examined using transmission electron microscopy (Hitachi 7,500, Tokyo, Japan). The thickness of the GBM and the width of the podocyte foot process were measured and calculated as previously described in the literature (19).

### Western blot analysis

Cortical kidney specimens from mice were placed on ice and lysed using RIPA buffer. Protein concentrations were determined using the bicinchoninic acid protein assay kit (Pierce Thermo-Scientific, Rockford, IL). Samples with equal amounts of total protein (50 μg/mL) were separated by 12% SDS–PAGE under reducing conditions and subjected to Western blot (WB) analysis. The membranes were incubated overnight at 4°C with primary antibodies against WT1 (Abcam Cat# ab89901; 1:1000), ZO-1 (GeneTex Cat# GTX636399; 1:1000), Col1a1 (Cell Signaling Technology Cat# 72026; 1:2000), snail1 (Abcam Cat# ab53519; 1:1000), β-catenin (Abcam Ca# ab32572; 1:1000) and α-tubulin (Abcam ca# ab7291; 1:2000), Subsequently, the membranes were incubated with horseradish peroxidase (HRP)-conjugated secondary antibodies for 1 h. Finally, the immunoreactive bands were visualized using an ECL kit (Amersham Biosciences, UK).

### Quantitative real-time PCR

Total RNA from renal cortex tissues was isolated using TRIzol reagent (Invitrogen cat# 15596018). Subsequently, the extracted RNA was reverse-transcribed into cDNA using a commercial kit (Vazyme Cat# R111-02). Gene expression levels were detected by real-time qRT‒PCR (Vazyme Cat# Q141–02) and an ABI7300 Sequence Detection System (Applied Biosystems, CA). The relative amount of mRNA, normalized against β-actin mRNA, was calculated as 2^-∆∆CT^. The primer sequences are listed in Table 1.

**Table 1:** The sequences of primers for qRT-PCR analysis.

Gene	Forward	Backward
Col1a1	GCTCCTCTTAGGGGCCACT	CCACGTCTCACCATTGGGG
WT1	GAGAGCCAGCCTACCATCC	GGGTCCTCGTGTTTGAAGGAA
ZO-1	GACTTGTCAGCTCAGCCA GT	GGCTCCTCTCTTGCCAACTT
β-actin	GGCTGTATTCCCCTCCATCG	CCAGTTGGTAACAATGCCATGT

WT1, Wilms tumor 1; ZO-1, zona occludens-1; Col1a1, collagen type I alpha I.

### Statistical analysis

All data are presented as the means ± SDs, comparisons of continuous variables between two groups were tested by using Student’s *t* tests, and comparisons of continuous variables among multiple groups were tested by using one-way ANOVA. Statistical analysis was executed using SPSS software version 22.0, and *p* < 0.05 was defined as statistically significant.

## Results

### Effects of KXC on blood and urine biochemical parameters in mice

**Figure 1:**
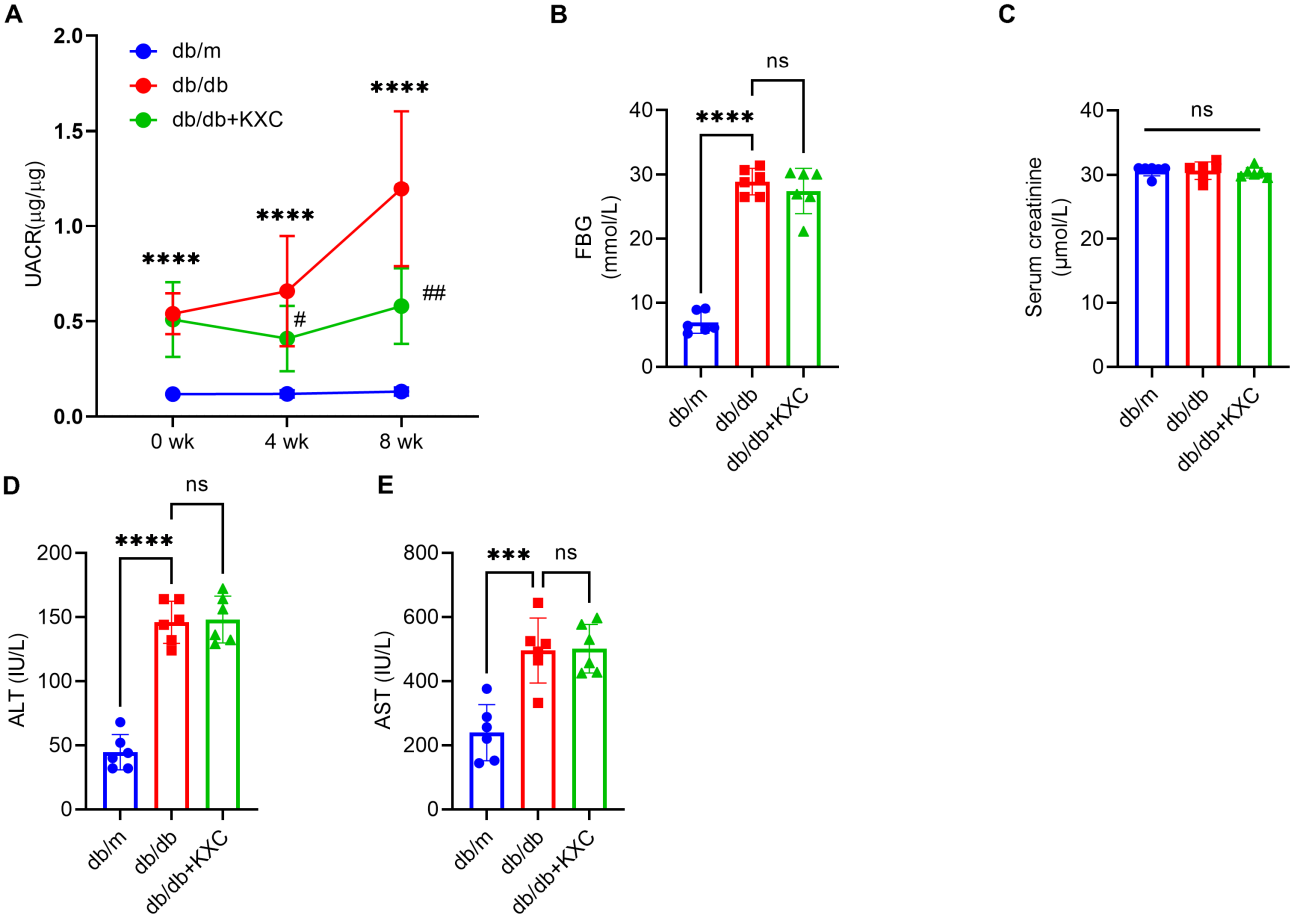
Effects of KXC treatment on the urinary albumin-to-creatinine ratio (UACR) and biochemical parameters in db/db mice. Changes in UACR **(A)**, FBG **(B)**, Scr **(C)**, ALT **(D)** and AST **(E)**. The results are expressed as the mean ± SD (n = 6). ***p < 0.001 and ****p < 0.0001 vs. db/m; #*p* < 0.05 and ##*p* < 0.01 vs. db/db.

We specifically chose 8-week-old db/db mice because they exhibit significant metabolic disturbances and increased albuminuria at this stage (20). Confirming our expectations, the db/db mice exhibited considerably higher levels of FBG and urinary UACR than the db/m group. During the 4-week treatment period, administration of KXC resulted in a marked reduction in UACR, and this therapeutic effect became more pronounced with extended treatment duration (Figure 1A). Interestingly, there were no noticeable differences in blood glucose levels between the KXC group and the untreated db/db group at the end of the treatment period (Figure 1B). Furthermore, we observed no remarkable variations in the levels of Scr (Figure 1C), ALT (Figure 1D), and AST (Figure 1E) between the KXC group and the untreated db/db group. These findings indicate that KXC administration effectively mitigated albuminuria in db/db mice with DKD, without affecting blood glucose levels or causing notable hepatotoxicity or renotoxicity.

### Effects of KXC on renal morphology

**Figure 2:**
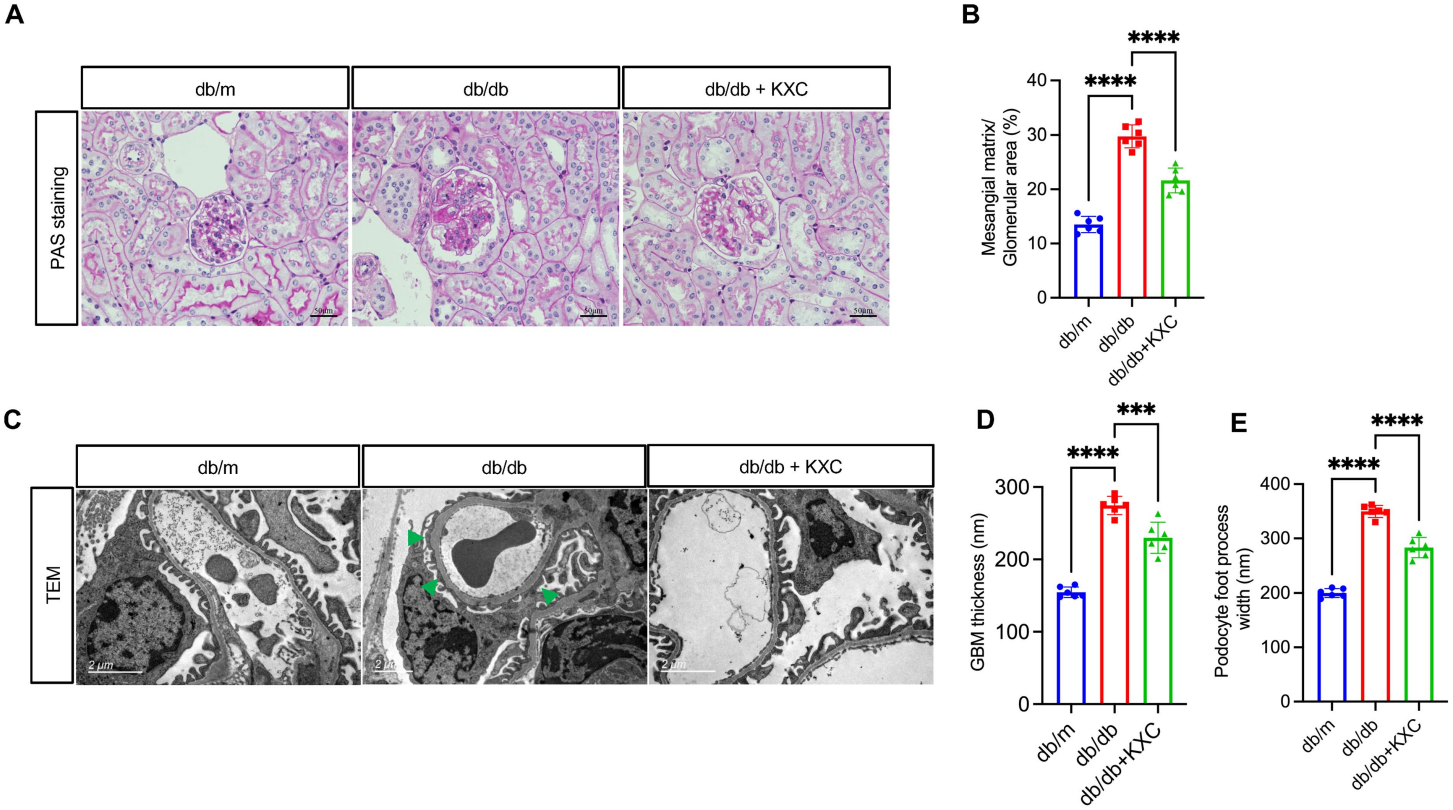
Effects of KXC treatment on renal morphology in db/db mice. **(A)** Representative PAS staining of kidney sections. **(B)** Semiquantitative analyses of the mesangial expansion index as shown by PAS staining. **(C)** Representative TEM images from each group of mice, scale bar=2 μm. Quantitative analysis of **(D)** GBM thickness and **(E)** podocyte foot process width. The results are expressed as the mean ± SD (*n* = 6). The arrow represents podocyte foot process effacement in db/db mice. ***p < 0.001, ****p < 0.0001.

Histological analysis of kidney sections stained with PAS revealed a remarkable expansion of the mesangial matrix in db/db mice compared to db/m mice, indicative of DKD. However, administration of KXC resulted in a marked amelioration of glomerular mesangial expansion, surpassing the observed levels in untreated db/db mice (Figures 2A, B). Furthermore, TEM examination of podocyte ultrastructure revealed notable thickening of the GBM and effacement of podocyte foot processes in db/db mice compared to db/m mice (Figures 2C, D). Interestingly, treatment with KXC prominently attenuated these histological changes, indicating its protective effects on podocyte structure and function. These findings hightlight the potential of KXC as a therapeutic intervention for DKD, specifically targeting mesangial matrix expansion and podocyte ultrastructural alterations.

### Therapeutic potential of KXC in modulating podocyte EMT

**Figure 3:**
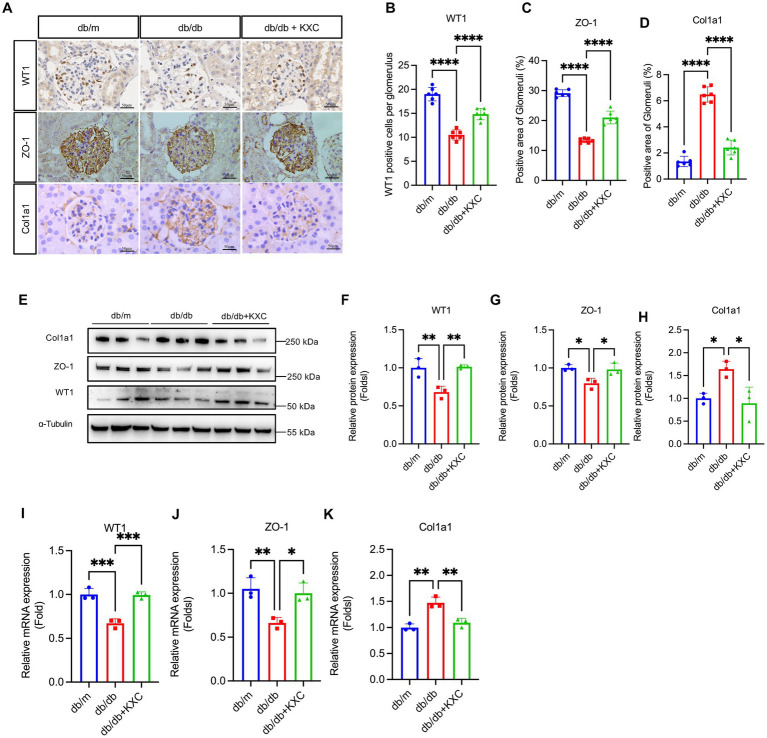
Expression of WT1, ZO-1 and Col1a1 in the kidneys of db/m mice and db/db mice with or without KXC treatment. **(A)** Representative images of WT1, ZO-1, and col1a1 stained by immunohistochemistry in the kidney sections. Semiquantitative analysis of immunostaining for the expression of glomerular **(B)** WT1, **(C)** ZO-1, and **(D)** col1a1 from db/m mice and db/db mice with or without KXC treatment. **(E)** Representative immunoblots and semiquantitative analysis of **(F)** WT1, **(G)** ZO-1, and **(H)** Col1a1 expression in the renal cortex from db/m mice and db/db mice with or without KXC treatment. mRNA expression of **(I)** WT1, **(J)** ZO-1, and **(K)** Col1a1 assessed using real-time PCR. The results are expressed as the mean ± SD (*n* = 6). *p < 0.05, **p < 0.01, ***p < 0.001, ****p < 0.0001.

Previous studies have consistently emphasized the pivotal role of podocyte EMT in the development of podocyte malfunction and proteinuria in DKD (21–26). To evaluate the effect of KXC on podocyte EMT, we utilized a comprehensive approach involving qPCR, WB analysis, and immunohistochemistry to assess the expression of key markers, including WT1, ZO-1, and Col1a1. Immunostaining revealed a significant decrease in the expression of WT1 and ZO-1 in db/db mice compared to db/m mice, indicating podocyte dysfunction in the diabetic state. Remarkably, KXC treatment led to a clear reversal of these decreases, suggesting its potential in restoring podocyte integrity and function. Moreover, we observed a notable increase in the expression of Col1a1 in db/db mice compared to db/m mice. However, this increase was significantly attenuated following KXC administration (Figures 3A–D). The immunohistochemical staining findings were further supported by WB and qPCR analyses, which consistently demonstrated alterations in the protein (Figures 3E–H) and mRNA (Figures 3I–K) expression levels of Col1a1, WT1, and ZO-1. Collectively, these results provide compelling evidence for the therapeutic potential of KXC in modulating podocyte EMT in DKD.

### Modulation of β-catenin signaling by KXC in mediating podocyte EMT

**Figure 4:**
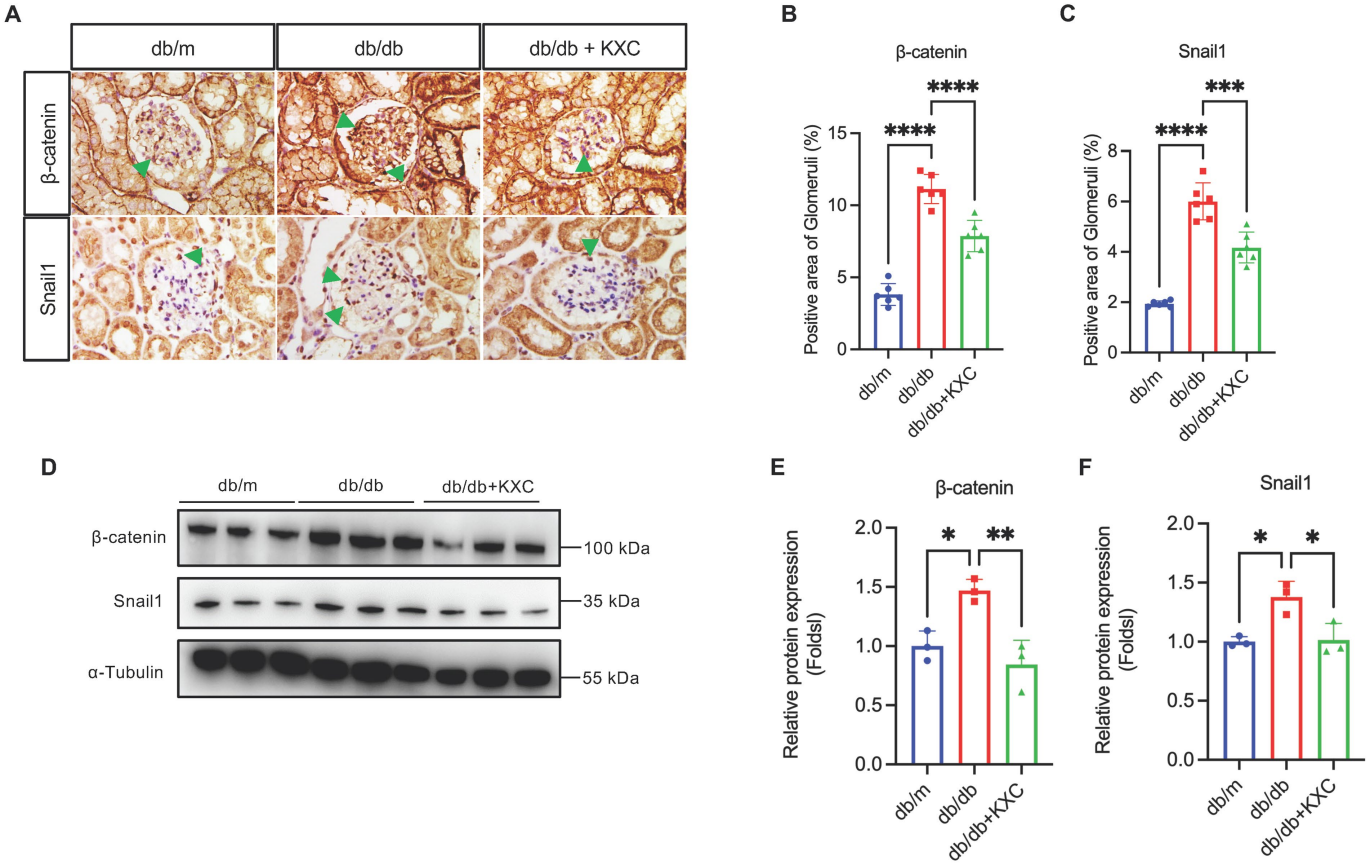
Expression of β-catenin and snail1 in the kidneys of db/m mice and db/db mice with or without KXC treatment. **(A)** Representative images of IHC staining of β-catenin and Snail1 in kidney sections. Semiquantitative analysis of immunostaining for glomerular **(B)** β-catenin and **(C)** snail1 expression in db/m mice and db/db mice with or without KXC treatment. **(D)** Representative immunoblotting analysis and semiquantitative analysis of **(E)** β-catenin and **(F)** snail1 expression in the renal cortex from db/m mice and db/db mice with or without KXC treatment. The results are expressed as the mean ± SD (*n* = 6). The arrow represents the protein expression of β-catenin and snail1 in db/db mouse glomeruli. *p < 0.05, **p < 0.01, ***p < 0.001, ****p < 0.0001.

Previous studies have extensively demonstrated the pivotal role of β-catenin signaling activation in mediating podocyte EMT and proteinuria (27–30). To assess the impact of KXC on β-catenin signaling, we evaluated the expression levels of β-catenin and snail1. Western blot (WB) analysis revealed a significant increase in β-catenin and snail1 protein expression in db/db mice. Notably, following KXC treatment, we observed a marked decrease in the expression of these proteins (Figures 4D–F). Furthermore, immunohistochemical staining provided additional confirmation of our results, illustrating the changes in β-catenin and snail1 expression (Figures 4A–C). This comprehensive analysis supported the notion that KXC treatment exerts a beneficial effect on β-catenin signaling, which may contribute to the preservation of podocyte integrity and the amelioration of proteinuria in DKD.

## Discussion

DKD has emerged as a significant global public health challenge (6, 31, 32). Proteinuria and declining kidney function are major clinical manifestations of DKD, serving as markers of disease severity and indicators to assess therapeutic effects (33, 34). Accumulating evidence supports podocyte injury as a pivotal factor in the occurrence of proteinuria and the progression of DKD (35, 36). Consequently, targeting podocytes has gained attention as an attractive strategy for developing novel treatments to prevent the progression of DKD.

This study provides evidence demonstrating the renoprotective effects of KXC on kidneys affected by diabetes. Our findings demonstrate significant improvement in the structural and functional abnormalities associated with DKD (including albuminuria excretion, glomerular mesangial matric accumulation, GBM thickening and podocyte foot process effacement) following KXC treatment. The underlying mechanisms may involve the alleviation of podocyte EMT through inhibition of β-catenin signaling. These results suggest that KXC has the potential to be a treatment option for preventing the progression of DKD.

Podocytes, highly specialized epithelial cells in mature kidneys with minimal proliferative capacity, are vulnerable to various injuries that result in dysfunction and loss. As essential functional cells, podocytes play a crucial role in maintaining the integrity of the glomerular filtration barrier (37). As mentioned earlier, proteinuria is a critical hallmark of DKD, and the relationship between podocyte injury and proteinuria has been extensively documented in both animal experiments and clinical studies (38–40). Several factors can contribute to podocyte injury (37). Emerging evidence highlights podocyte EMT as a key driver of podocyte dysfunction in DKD. Under diabetic conditions, podocytes can undergo phenotypic switching marked by the downregulation of epithelial markers (e.g., WT1, ZO-1, nephrin, and P-cadherin) and the upregulation of mesenchymal markers (e.g., desmin, Col1a1, and fibronectin). EMT enhances the motility of podocytes, resulting in their detachment from the GBM and ultimately impairing the integrity of renal filtration (21–23, 25, 26, 41). Therefore, inhibiting podocyte EMT represents a novel treatment option for DKD.

In this study, we observed increased protein and mRNA expression of Col1a1 and decreased expression of WT1 and ZO-1 in the glomeruli of db/db mice. These changes were reversed after 8 weeks of KXC treatment, which correlated with the improvement in UACR excretion and histological impairment in db/db mice. Based on our findings, we deduced that KXC may have a significant suppressive effect on podocyte EMT in db/db mice.

The mechanism underlying the renoprotective effects of KXC, particularly in alleviating podocyte EMT in DKD, has received limited investigation. Emerging evidence demonstrates that the Wnt/β-catenin pathway plays a significant role in facilitating podocyte EMT (27). Moreover, numerous previous studies have consistently shown the activation of the Wnt/β-catenin pathway in podocytes of both diabetic patients and experimental mouse models (27–29, 42). Activation of this pathway leads to the nuclear translocation of β-catenin and subsequent transcriptional upregulation of downstream target genes (43). Snail1, a critical downstream target gene of β-catenin, plays a crucial role in expediting podocyte EMT (44–46). Studies have shown that the activation of β-catenin leads to upregulated expression of snail1 in podocytes, which induces podocyte EMT in DKD (27, 47). Hence, targeting the Wnt/β-catenin pathway could be a promising protective approach for alleviating podocyte EMT in DKD. Our findings demonstrated that the expression of β-catenin and snail1 in the glomeruli was significantly increased, which was reversed after KXC treatment. These findings suggest that the alleviation of podocyte EMT by KXC may be partially attributed to the inhibition of Wnt/β-catenin pathway activation.

KXC has been proven to be a safe medication in numerous previous clinical studies (12–16). Consistently, no noticeable liver or kidney toxicity was observed in our animal model, as confirmed by the lack of influence on the levels of AST, ALT and Scr. Additionally, our study revealed no apparent impact of KXC on blood sugar levels, suggesting that the renoprotective effects of KXC may be independent of its hypoglycemic effects.

There was a limitation in our study. Since KXC is a TCM with multiple components and targets, additional research is required to elucidate other potential mechanisms underlying its protective effects against podocyte injury in DKD.

In summary, our study demonstrated that KXC has the potential to protect podocytes during DKD by alleviating podocyte EMT through inactivating β-catenin signaling. These results suggest that KXC could be a promising novel therapeutic agent for preventing the progression of DKD. Additional clinical trials should be conducted to confirm its safety and effectiveness among individuals with DKD.

## Data availability statement

The raw data supporting the conclusions of this article will be made available by the authors, without undue reservation.

## Author contributions

BJ and CJ supervised and designed this project. BJ, JLiu, and YZ wrote the manuscript, performed *in vivo* experiments, and contributed to the data interpretation. JLu and QZ performed histologic assessment. YL and QS performed statistical analyses. All authors contributed to the article and approved the submitted version.

## Funding

This work was supported by the Key Project Supported by the Medical Science and Technology Development Foundation, Nanjing Department of Health (YKK21094).

## Conflict of interest

The authors declare that the research was conducted in the absence of any commercial or financial relationships that could be construed as a potential conflict of interest.

## Publisher’s note

All claims expressed in this article are solely those of the authors and do not necessarily represent those of their affiliated organizations, or those of the publisher, the editors and the reviewers. Any product that may be evaluated in this article, or claim that may be made by its manufacturer, is not guaranteed or endorsed by the publisher.

## Supplementary material

The Supplementary material for this article can be found online at:

https://www.frontiersin.org/articles/10.3389/fmed.2023.1213191/full#supplementary-material
