# Supplementary figures and images for: Kunxian capsule alleviates podocyte injury and proteinuria by inactivating β-catenin in db/db mice
Source: Front Med (Lausanne). 2023 Jun 30;10:1213191. doi: 10.3389/fmed.2023.1213191 (PMC10349331; doi:10.3389/fmed.2023.1213191)

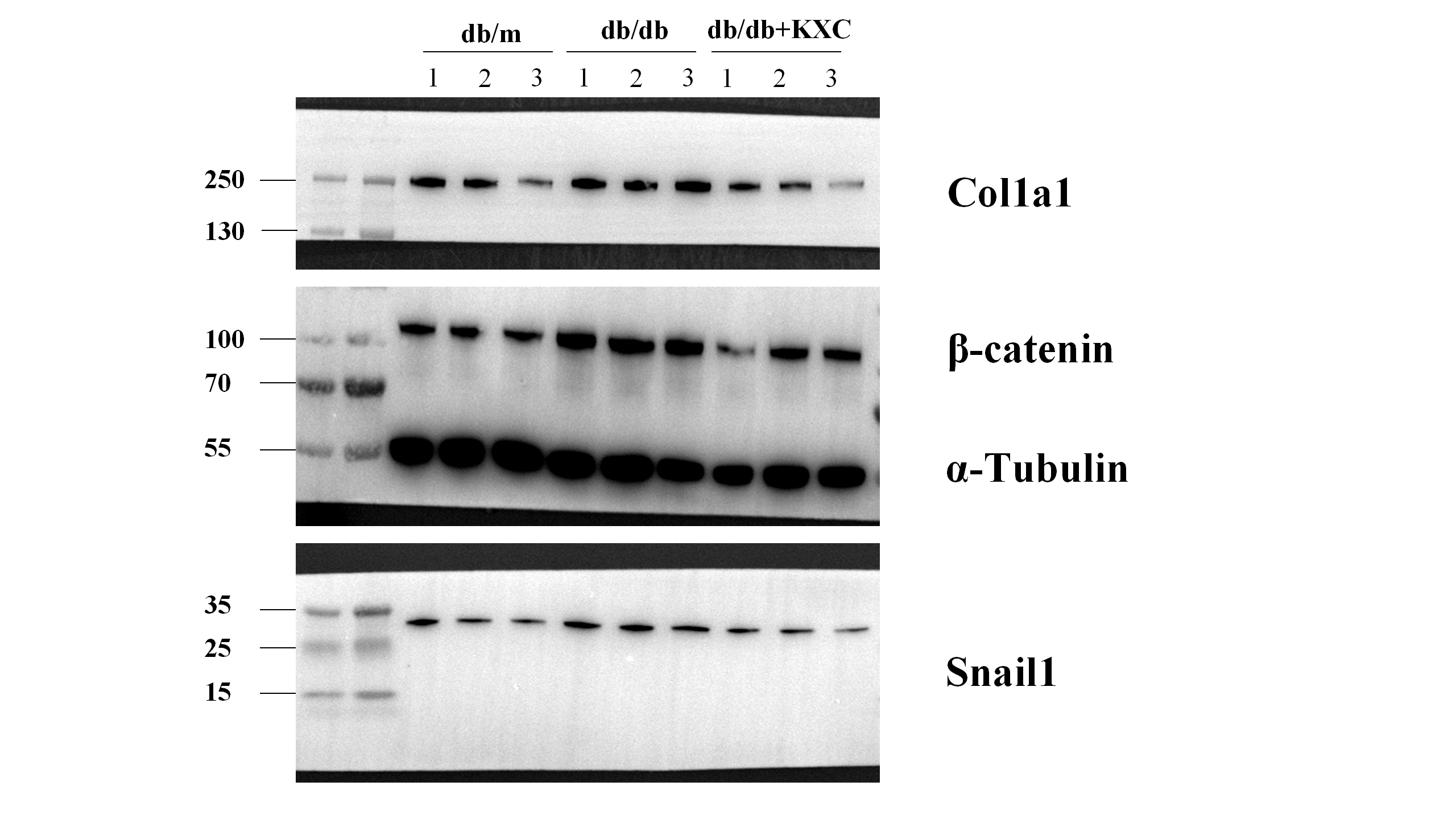

Supplement: Supplementary file 1 [file Image_1.TIF]

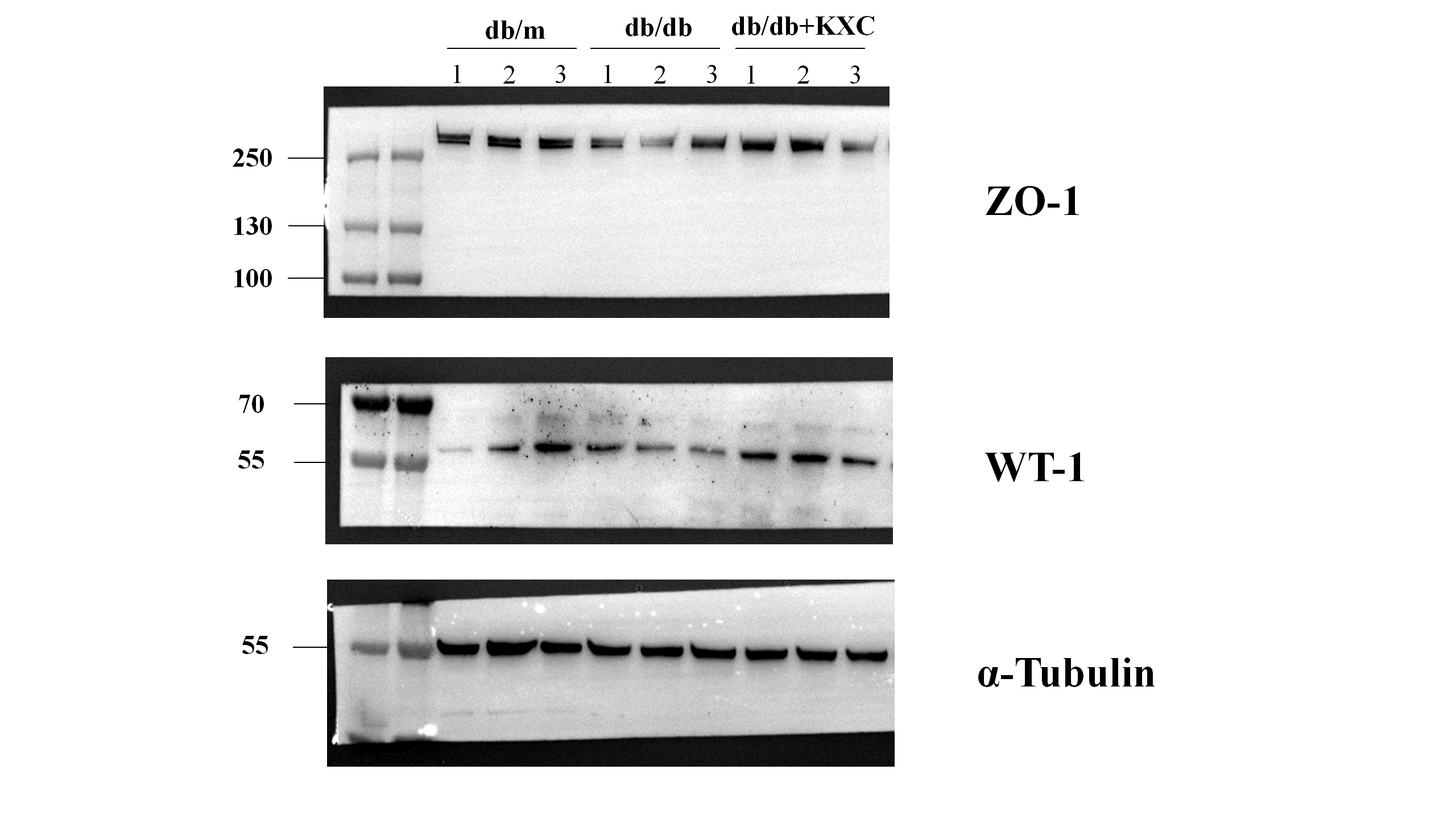

Supplement: Supplementary file 2 [file Image_2.TIF]
